# Sedentary Behavior Is Only Marginally Associated with Physical Function in Adults Aged 40–75 Years—the Maastricht Study

**DOI:** 10.3389/fphys.2017.00242

**Published:** 2017-04-25

**Authors:** Jeroen H. P. M. van der Velde, Hans H. C. M. Savelberg, Julianne D. van der Berg, Simone J. S. Sep, Carla J. H. van der Kallen, Pieter C. Dagnelie, Miranda T. Schram, Ronald M. A. Henry, Petronella L. M. Reijven, Tineke A. C. M. van Geel, Coen D. A. Stehouwer, Annemarie Koster, Nicolaas C. Schaper

**Affiliations:** ^1^Department of Human Movement Sciences, Maastricht UniversityMaastricht, Netherlands; ^2^NUTRIM School for Nutrition and Translational Research in Metabolism, Maastricht UniversityMaastricht, Netherlands; ^3^Division of Endocrinology, Department of Internal Medicine, Maastricht University Medical CentreMaastricht, Netherlands; ^4^CARIM School for Cardiovascular Diseases, Maastricht UniversityMaastricht, Netherlands; ^5^Department of Social Medicine, Maastricht UniversityMaastricht, Netherlands; ^6^CAPHRI Care and Public Health Research Institute, Maastricht UniversityMaastricht, Netherlands; ^7^Radboud Institute for Health Sciences, Radboud University Medical CenterNijmegen, Netherlands; ^8^Department of Internal Medicine, Maastricht University Medical CentreMaastricht, Netherlands; ^9^Department of Epidemiology, Maastricht UniversityMaastricht, Netherlands; ^10^Heart and Vascular Centre, Maastricht University Medical CentreMaastricht, Netherlands; ^11^Department of Dietetics, Maastricht University Medical CentreMaastricht, Netherlands; ^12^Department of Family Medicine, Maastricht UniversityMaastricht, Netherlands

**Keywords:** accelerometry, muscle strength, sedentary lifestyle, pattern, physical fitness

## Abstract

**Background:** In an aging population, regular physical activity (PA) and exercise have been recognized as important factors in maintaining physical function and thereby preventing loss of independence and disability. However, (older) adults spent the majority of their day sedentary and therefore insight into the consequences of sedentary behavior on physical function, independent of PA, is warranted.

**Objective:** To examine the associations of objectively measured sedentary time (ST), patterns of sedentary behavior, overall PA, and higher intensity PA (HPA) with objective measures of physical function.

**Methods:** This is a cross-sectional study in 1,932 men and women (aged 40–75 years) participating in The Maastricht Study. The activPAL3 was used to assess daily sedentary behavior: ST (h), sedentary breaks (n), prolonged (≥30 min) sedentary bouts (n), and to assess time spent in (H)PA (h). Measures of physical function included: covered distance during a 6 min walk test [6MWD (meters)], timed chair rise stand test performance [TCST_time_ (seconds)], grip strength (kg kg^−1^), and elbow flexion and knee extension strength (Nm kg^−1^). Linear regression analyses were used to examine associations between daily sedentary behavior and PA with physical function.

**Results:** Every additional hour ST was associated with shorter 6MWD [B = −2.69 m (95% CI = −4.69; −0.69)] and lower relative elbow extension strength (B = −0.01 Nm kg^−1^ (−0.02; 0.00). More sedentary breaks were associated with faster TCST_time_: B = −0.55 s (−0.85; −0.26). Longer average sedentary bout duration was associated with slower TCST_time_ [B = 0.17 s (0.09; 0.25)] and lower knee extension strength [B = −0.01 Nm kg^−1^ (−0.02; 0.00)]. Every hour of PA and HPA were associated with greater 6MWD [B_PA_ = 15.88 m (9.87; 21.89), B_HPA_ = 40.72 m (30.18; 51.25)], faster TCST_time_ [B_PA_ = −0.55 s (−1.03; −0.07), B_HPA_ = −2.25 s (−3.09; −1.41)], greater elbow flexion strength [B_PA_ = 0.03 Nm kg^−1^ (0.01; 0.07)], [B_HPA_ = 0.05 Nm kg^−1^ (0.01; 0.08)], and greater knee extension strength [B_PA_ = 0.04 Nm kg^−1^ (0.01; 0.07)], [B_HPA_ = 0.13 Nm kg^−1^ (0.06; 0.20)].

**Conclusion:** In adults aged 40–75 years, sedentary behavior appeared to be marginally associated with lower physical function, independent of HPA. This suggests that merely reducing sedentary behavior is insufficient to improve/maintain physical function. In contrast, engaging regularly in PA, in particular HPA, is important for physical function.

## Introduction

Physical function, or physical capability, can be defined as the degree to which a person can manage the physical tasks of daily living. This can be objectified by several performance tests such as strength, walking speed, and mobility. Deterioration in physical function has been associated with loss of independence, a reduced quality of life, disability, and mortality (Cooper et al., 2010, 2011). Limitations in physical functioning occur more often in later stages of life. For example, in the European Union 27% of the total population reported limitations in daily activities, for adults aged >65 years this was ~40%, and for adults aged >75 years in excess of 60% (Statistical Office of the European Communities, 2015). In an aging population, such as in many European countries, the number of people at risk for functional limitations will increase further. Thus, identifying modifiable determinants that are important for improving or maintaining physical function is imperative. One of these determinants is physical activity (PA; Paterson and Warburton, 2010).

PA, particularly PA of higher intensity, often termed moderate to vigorous PA (MVPA), has been recognized as a major determinant for overall physical well-being (Warburton et al., 2010). Positive associations of MVPA with physical function (Paterson and Warburton, 2010; Bauman et al., 2016) and with leg strength (Volkers et al., 2012) have been reported. The importance of MVPA is nowadays well-recognized and PA guidelines worldwide advocate to spend at least 150 min per week in MVPA (Kahlmeier et al., 2015). Nonetheless, MVPA only comprises a small part of daily activities. Most of the day is generally spent in sedentary behavior in current Westernized societies (Owen et al., 2010). In recent years, there has been a growing interest in sedentary behavior as a determinant for adverse health outcomes.

Sedentary behavior refers to any waking behavior, characterized by an energy expenditure ≤ 1.5 metabolic equivalents (METs) while in a sitting or reclining position (Sedentary Behaviour Research Network, 2012). An increasing number of studies have associated a larger amount of sedentary time (ST) with unfavorable metabolic and cardiovascular risk markers, independent of MVPA (Wilmot et al., 2012; Brocklebank et al., 2015). However, whether or not a larger amount of ST is associated with lower physical function is less clear. Several population based studies have examined the association of ST and physical function (Santos et al., 2012; Cooper et al., 2015; Lee et al., 2015; Keevil et al., 2016; Reid et al., 2016; Rosenberg et al., 2016). Findings from these studies were inconsistent as some studies did report an association between larger amounts of ST and worse physical function (Santos et al., 2012; Cooper et al., 2015; Lee et al., 2015; Rosenberg et al., 2016), whereas other studies reported no such association (Keevil et al., 2016; Reid et al., 2016). Additionally, not only total ST, but also the pattern in which it is accumulated may be relevant for health. This pattern can be expressed by the number of interruptions in ST (sedentary breaks), by the average duration of uninterrupted periods of sitting or by the number of prolonged (e.g., ≥30 min) uninterrupted sedentary bouts. In studies with older adults (mean age >70 years), more sedentary breaks have been associated with a higher score on the senior fitness test and physical performance tests (Davis et al., 2014; Sardinha et al., 2015). Whether or not these patterns are associated with physical function at younger ages is uncertain.

As sedentary behavior appears to increase with age (Matthews et al., 2008; Evenson et al., 2012), an improved insight into the associations of ST (and the pattern in which this is accumulated) with physical function is warranted. If such associations exist, reducing sedentary behavior could be important in the prevention of functional limitations. Therefore, our objective was to examine the associations of objectively measured ST, patterns of sedentary behavior, overall PA, and higher intensity PA (HPA) with objective measures of physical function in an adult population aged 40–75 years.

## Methods

### Population

We used data from The Maastricht Study, an observational prospective population-based cohort study. The rationale and methodology have been described previously (Schram et al., 2014). In brief, the study focuses on the etiology, pathophysiology, complications and comorbidities of type 2 diabetes mellitus (T2DM) and is characterized by an extensive phenotyping approach. Eligible for participation were all individuals aged between 40 and 75 years and living in the southern part of the Netherlands. Participants were recruited through mass media campaigns and from the municipal registries and the regional Diabetes Patient Registry via mailings. Recruitment was stratified according to known T2DM status, with an oversampling of individuals with T2DM, for reasons of efficiency. The present report includes cross-sectional data from a convenience sample of the first 3,451 participants, who completed the baseline survey between November 2010 and September 2013. Data were available for 1,932 participants, after excluding participants that did not receive an accelerometer due to logistics (*n* = 673), with invalid accelerometer readings (*n* = 136), with missing/unperformed physical function testing (*n* = 629) or with missing covariates (*n* = 81). The examinations of each participant were performed within a time window of 3 months. The study has been approved by the institutional medical ethical committee (NL31329.068.10) and the Minister of Health, Welfare and Sports of the Netherlands (Permit 131088-105234-PG). All participants gave written informed consent in accordance with the Declaration of Helsinki.

### Accelerometry: sedentary behavior, PA, and HPA

Daily activity levels were measured using the activPAL3™ PA monitor (PAL Technologies, Glasgow, UK). The activPAL3 is a small (53 × 35 × 7 mm), lightweight (15 g) triaxial accelerometer that records movement in the vertical, anteroposterior and mediolateral axes, and also determines posture (sitting or lying, standing, and stepping) based on acceleration information. The device was attached directly to the skin on the front of the right thigh with transparent 3M Tegaderm™ tape, after the device had been waterproofed using a nitrile sleeve. Participants were asked to wear the accelerometer for 8 consecutive days, without removing it at any time. To avoid inaccurately identifying non-wear time, participants were asked not to replace the device once removed. Data were uploaded using the activPAL software and processed using customized software written in MATLAB R2013b (MathWorks, Natick, MA, USA). Data from the first day were excluded from the analysis because participants performed physical function tests at the research center after the device was attached. In addition, data from the final wear day providing ≤ 14 waking hours of data were excluded from the analysis. Participants were included if they provided at least 1 valid day (≥10 h of waking data).

The total amount of ST was based on the sedentary posture (sitting or lying), and calculated as the mean time spent in a sedentary position during waking time per day. The method used to determine waking time has been described elsewhere (van der Berg et al., 2016). The total amount of standing time was based on the standing posture, and calculated as the mean time spent standing during waking time per day. The total amount of stepping was based on the stepping posture, and calculated as the mean time stepping during waking time per day. Stepping time (PA) was further classified into higher intensity physical activity (HPA; minutes with a step frequency >110 steps/min during waking time) and lower intensity physical activity (LPA; minutes with a step frequency ≤ 110 steps/min during waking time; Tudor-Locke et al., 2011).

The number of sedentary breaks during waking time was determined as each transition from a sitting or lying position to standing or stepping with a duration of at least 1 min, and the mean number of breaks per day was calculated. ST accumulated in a consecutive period ≥30 min was defined as a prolonged sedentary bout, and the mean number of prolonged sedentary bouts during waking time per day was calculated.

### Physical function

Physical function was assessed by four different tests: a fast paced 6 min walk test, the timed chair stand test (TCST), hand grip strength, and isometric strength tests of the knee extensors and elbow flexors.

#### Six minute walk test

Participants were excluded from this test if they had experienced cardiovascular complications in the preceding 3 months, had severe hypertension (SBP ≥ 180 and/or DBP ≥ 110 mmHg), a resting heart rate of <40 of >110 beats min^−1^, used a walker, or had other medical conditions which prevented them from walking independently. In a hallway, two cones were placed 20 meters apart around which the participants had to make turns. Participant were instructed to walk as many laps as possible in 6 min at a fast pace without running. Standardized encouragement was given every minute during the test. After 6 min, or when the participant was unwilling or unable to continue, the covered distance was measured. The covered distance (6MWD) in meters was used as measure for analyses.

#### Timed chair rise stand test

The timed chair rise stand test (TCST) was performed on a 46 cm high chair with a straight back and no arm-rests. The test started with the participant in a sitting position with his/her arms crossed over the chest. Participants were instructed to stand up to a full up-right position and to sit down again, as quickly as possible, without using their arms or hands to support. The time (in seconds) needed for 10 repetitions (TCST_time_) was measured to the nearest of one decimal and was used for analyses.

#### Handgrip strength

Handgrip strength was measured with the Jamar handheld dynamometer (SEHAN Corp., Korea-Biometrics Europe BV, Almere). During the test the participant was standing straight against the wall, with the upper arm along the trunk and the elbow in 90° flexion. Participants were instructed to squeeze as hard as possible in the dynamometer for 3–5 s, while given standard encouragement. The measurement was performed three times on each hand, alternating hands. Maximal strength (kg) from every trial was recorded. Maximum strength (in kg) out of all trials was normalized for body mass and was used for analyses.

#### Isometric muscle strength test

Isometric muscle strength of the knee extensors and elbow flexors was assessed in a customized set-up with 2 dynamometers (Futek LSB302, FUTEK Advanced Sensor Technology Inc., Irvine, CA, USA) and recorded with the M-PAQ (Maastricht Instruments, Maastricht, the Netherlands). Measurements were performed on the right leg and arm. Participants were (partly) excluded from the test if they had undergone surgery on the right arm or leg in the preceding 3 months, or reported relevant injuries on the right arm or leg.

Participants were positioned up-right in the chair (hip angle 110°) with their knees flexed in a 90° angle and the upper leg fixated. A strap connected to the dynamometer (with the axis of the dynamometer corresponding to the knee-joint axis) was secured 2 cm above the lateral malleolus. Participants were instructed to extend their knee as powerful as possible for 5 s. Three trials were performed. For the measurement of elbow flexion strength, the participant remained up-right in the chair with the elbow flexed in a 90° angle. A strap connected to the dynamometer was secured 2 cm proximally from the wrist (with the axis of the dynamometer corresponding to the elbow-joint axis). Participants were instructed to flex their elbow as powerful as possible for 5 s. Three trials were performed. Participants were able to see the force generated on a monitor. During the trials participants were instructed to refrain from compensatory movements.

To calculate joint torques (Nm) for elbow and knee, the force applied on the dynamometers (N) was multiplied by the corresponding moment-arm (distance from the strap of the dynamometer to the rotation point of the knee joint and elbow joint, respectively). The joint torques were normalized for body mass (Nm/kg). The maximal normalized joint torques out of three trials for knee extension and elbow flexion were used in the analyses.

### Covariates

Questionnaires were conducted to collect information on age (in years), sex, educational level, smoking behavior, alcohol consumption, cardiovascular disease history (CVD), self-reported physical functioning, and health status. Educational level was divided into low, middle, and high. Smoking behavior was divided into three categories: non-smoker, former smokers, and current smokers. Alcohol consumption was divided into three categories: non-consumers, low-consumers (for women ≤ 7 glasses alcohol per week; for men ≤ 14 glasses alcohol per week), and high-consumers (for women >7 glasses per week; for men >14 glasses alcohol per week). CVD was defined as a (self-reported) history of any of the following conditions: myocardial infarction, cerebrovascular infarction or hemorrhage, percutaneous artery angioplasty of, or vascular surgery on, the coronary, abdominal, peripheral, or carotid arteries. Self-reported physical functioning was based on the physical function score, ranging from 0 to 100, as obtained from the 36-Item Short Form Health Survey (SF-36). Health status was obtained from self-reported general health status on a 5-point scale ranging from “weak” to “excellent.” BMI was calculated as: body mass (kg)/height (m)^2^. For this, mass and height were measured to the nearest of 0.5 kg or 0.1 cm during physical examination. Type 2 diabetes was defined according to the World Health Organization 2006 criteria (World Health Organization, 2006), based on glucose levels in fasting state and directly after an oral glucose tolerance test. For details on this procedure see Schram et al. (2014).

### Statistical analyses

Descriptive statistics were presented for the included population and according to sex. Normally distributed variables were presented as mean (*SD*), skewed variables were presented as median [25–75%]. Percentages were provided for categorical variables.

Linear regression analyses were performed to assess the associations of ST, number of sedentary breaks, average sedentary bout duration, and number of prolonged sedentary bouts, total PA and HPA with the physical function measures. Associations were expressed as regression coefficients (B) with 95% confidence intervals. The associations in models 1 were adjusted for waking time, age, sex, education level, and type 2 diabetes (to account for oversampling in the study design). To assess if the associations were mutually independent, in models 2 HPA, was added to the models describing ST, ST was added in the models describing the associations of HPA (due to collinearity models of total PA were not adjusted for ST), and ST and HPA were both added in the models describing sedentary breaks, mean sedentary bout duration and number of prolonged sedentary bouts. Models 3 were additionally adjusted for several health-related factors: BMI, alcohol use, smoking status, CVD history, and health status. We chose to add these health-related factors in models 3 as some of these factors may cause overadjustment bias (in particular BMI and health status). For the ease of interpretation we chose to express the associations of ST, total PA and HPA per 1 h. In additional analyses, we have standardized these three exposure variables to allow a better comparison of strengths of the associations. Additionally, the analyses were repeated after excluding all participants with <4 valid days of activPAL data (*n* = 78) and after excluding participants who reported functional limitations, defined as having difficulty walking 500 m or climbing one flight of stairs as reported on the SF-36 (*n* = 328). All analyses were performed using IBM SPSS Statistics for Windows, Version 22.0. (Armonk, NY, USA: IBM Corp.).

## Results

### Population characteristics

Of the 1,932 participants, 51.4% were men. The mean (±*SD*) age was 59.7 (8.2) years and BMI was 26.8 (4.4; Table 1). In over 95% of participants 4 or more days with valid accelerometer data were obtained. During waking time 9.4 (1.6) h/day were spent in sedentary positions and 2.0 (0.7) h/day were spent in PA. The remainder of time was spent standing. Women spent less time in sedentary behavior and more time in HPA than men. Mean TCST_time_ was similar between men and women. Mean 6MWD and strength measures were greater for men compared with women. When strength measures were adjusted for body mass the differences between sexes were reduced, but relative measures of strength were still greater in men.

**Table 1:** **Descriptive characteristics of the study population (***N*** = 1,932)**.

	**Total population**		**Men (*****n*** = **993)**		**Women (*****n*** = **939)**	
Age	59.7	(8.2)	60.8	(8.1)	58.6	(8.1)
Educational level (% high)	39.3		43.5		34.9	
Smoking status (% current)	12.5		13.5		11.5	
Alcohol consumption (% high)	26.3		23.7		29.2	
BMI	26.8	(4.4)	27.5	(4.0)	26.1	(4.6)
History of CVD (%)	15.7		18.8		12.4	
Type 2 diabetes mellitus (%)	26.0		36.0		15.5	
SF-36 physical function score	95	[85–100]	95	[85–100]	95	[80–100]
Valid days accelerometer data (n)	6.3	1.2	6.3	1.2	6.4	1.1
Waking time (h/day)	15.7	(0.9)	15.8	(0.9)	15.7	(0.9)
Sedentary time (h/day)	9.4	(1.6)	9.9	(1.5)	8.8	(1.6)
Total PA (h/day)	2.0	(0.7)	2.0	(0.7)	2.1	(0.6)
High intensity PA (min/day)	19.2	[9.6–32.0]	14.1	[6.9–26.5]	23.6	[14.5–35.7]
Sedentary breaks (N/day)	37.6	(8.5)	37.7	(9.0)	37.5	(8.0)
Average sedentary bout duration (min)	11.1	3.4	11.8	(3.7)	10.4	(2.9)
Sedentary bouts ≥30 min (N/day)	4.8	(1.5)	5.1	(1.6)	4.5	(1.4)
6 MWD (m)	585.1	(80.5)	594.1	(86.0)	575.5	(73.0)
Timed chair stand test (s)	23.8	(5.5)	23.8	(5.7)	23.7	(5.2)
Grip strength (kg)	35.7	(10.6)	43.6	(8.1)	27.4	(5.4)
Normalized grip strength (kg kg^−1^)	0.45	(0.12)	0.50	(0.11)	0.39	(0.09)
Elbow flexion strength (Nm)	59.2	(23.5)	73.2	(21.4)	44.2	(14.5)
Normalized elbow flexion (Nm kg^−1^)	0.75	(0.27)	0.86	(0.26)	0.64	(0.22)
Knee extension strength (Nm)	134.9	(44.8)	161.5	(39.8)	106.9	(30.4)
Normalized knee extension (Nm kg^−1^)	1.72	(0.48)	1.88	(0.46)	1.54	(0.45)

*BMI, body mass index; CVD, cardiovascular disease; PA, physical activity; 6MWD, distance covered during six min walk test. Values expressed as mean (SD), median [25–75%], or percentages*.

### Sedentary time and patterns of sedentary behavior and physical function

Table 2 describes the associations of the sedentary behavior variables (sedentary time, sedentary breaks, average sedentary bout duration, and prolonged sedentary bouts) with measures of physical function. An additional hour of ST was associated with shorter 6MWD [B = −2.69 m (95% CI = −4.69; −0.69)] and lower elbow flexion strength [B = −0.01 Nm kg^−1^ (−0.02; 0.00)] independent of HPA and other potential confounders (model 3). Every 10 additional sedentary breaks per day were associated with better TCST_time_ [B = −0.55 s (−0.85; −0.26)] in model 3, but not with the other measures of physical function. A longer average sedentary bout duration was associated with poorer performance on the TCST_time_ [B = 0.17 s (0.09; 0.25)] and with lower relative knee extension strength [B = −0.01 Nm kg^−1^ (−0.02; 0.00)].

**Table 2:** **Associations of sedentary time and sedentary behavior pattern variables with distance during a six min walk test (6WMD), timed chair rise stand test performance (TCST time), grip strength, elbow flexion strength, and knee extension strength**.

		**Model 1**		**Model 2**		**Model 3**	
		**B**	**95% CI**	**B**	**95% CI**	**B**	**95% CI**
Sedentary time (h/day)	6 MWT distance (m)	**−7.46**	**(−9.51; −5.40)**	**−4.39**	**(−6.49; −2.29)**	**−2.69**	**(−4.69; −0.69)**
	TCST time (s)^*^	**0.30**	**(0.15; 0.46)**	**0.16**	**(0.00; 0.32)**	0.11	(−0.05; 0.27)
	Grip strength (kg kg^−1^)	**−0.01**	**(−0.01; −0.01)**	**−0.01**	**(−0.01; 0.00)**	0.00	(−0.01; 0.00)
	Elbow flexion strength (Nm kg^−1^)	**−0.02**	**(−0.03; −0.01)**	**−0.02**	**(−0.02; −0.01)**	**−0.01**	**(−0.02; 0.00)**
	Knee extension strength (Nm kg^−1^)	**−0.03**	**(−0.04; −0.01)**	**−0.02**	**(−0.03; 0.00)**	−0.01	(−0.02; 0.01)
Sedentary breaks (10/day)	6 MWT distance (m)	**8.46**	**(4.53; 12.39)**	**5.32**	**(1.46; 9.18)**	2.57	(−1.13; 6.28)
	TCST time (s)^*^	**−0.71**	**(−1.00; −0.42)**	**−0.59**	**(−0.88; −0.30)**	**−0.55**	**(−0.85; −0.26)**
	Grip strength (kg kg^−1^)	**0.01**	**(0.01; 0.02)**	**0.01**	**(0.00; 0.01)**	0.00	(−0.01; 0.01)
	Elbow flexion strength (Nm kg^−1^)	**0.03**	**(0.01; 0.04)**	**0.02**	**(0.01; 0.03)**	0.01	(−0.01; 0.02)
	Knee extension strength (Nm kg^−1^)	**0.05**	**(0.02; 0.07)**	**0.04**	**(0.01; 0.06)**	0.02	(−0.01; 0.04)
Average sedentary bout duration (min)	6 MWT distance (m)	**−3.46**	**(−4.41; −2.52)**	**−1.81**	**(−2.89; −0.72)**	−0.68	(−1.74; 0.37)
	TCST time (s)^*^	**0.22**	**(0.15; 0.29)**	**0.18**	**(0.10; 0.26)**	**0.17**	**(0.09; 0.25)**
	Grip strength (kg kg^−1^)	**−0.01**	**(−0.01; 0.00)**	**−0.00**	**(−0.01; 0.00)**	0.00	(0.00; 0.00)
	Elbow flexion strength (Nm kg^−1^)	**−0.01**	**(−0.01; −0.01)**	**−0.01**	**(−0.01; 0.00)**	0.00	(−0.01; 0.00)
	Knee extension strength (Nm kg^−1^)	**−0.02**	**(−0.03; −0.01)**	**−0.02**	**(−0.02; −0.01)**	**−0.01**	**(−0.02; 0.00)**
≥30 min sedentary bout (n/day)	6 MWT distance (m)	**−7.75**	**(−9.83; −5.68)**	**−4.08**	**(−7.36; −0.79)**	−2.85	(−5.98; 0.28)
	TCST time (s)^*^	**0.34**	**(0.18; 0.49)**	0.23	(−0.24; 0.48)	0.21	(−0.04; 0.46)
	Grip strength (kg kg^−1^)	**−0.01**	**(−0.01; −0.01)**	**−0.01**	**(−0.01; 0.00)**	0.00	(−0.01; 0.00)
	Elbow flexion strength (Nm kg^−1^)	**−0.02**	**(−0.03; −0.01)**	−0.01	(−0.02; 0.00)	0.00	(−0.01; 0.01)
	Knee extension strength (Nm kg^−1^)	**−0.03**	**(−0.04; −0.02)**	**−0.02**	**(−0.04; 0.00)**	−0.01	(−0.03; 0.01)

*Results are presented as unstandardized regression coefficients (B) with 95% confidence interval (95% CI)*.

* *Positive coefficient indicates poorer performance. Associations were adjusted for the following covariates; Model 1: waking time, age, sex, type 2 diabetes, and education level. Model 2: model 1 + HPA. Model 3: model 2 +BMI, alcohol use, smoking status, cardiovascular disease, and health status. Bold fonts indicate statistical significance (p < 0.05)*.

### Physical activity and physical function

Table 3 describes the associations of total PA and HPA with measures of physical function. Total PA was associated with all the different physical function outcome measures in models 1. After additional adjustment for BMI, alcohol use, smoking status, cardiovascular disease, and health status (models 3) an additional hour of total PA was statistically significant associated with longer 6MWD [B = 16.45 m (11.89; 21.02)], better TCST_time_ [B = −0.67 s (−1.03; −0.30)], and greater elbow flexion strength [B = 0.03 Nm kg^−1^ (0.01; 0.07)] and knee extension strength [B = 0.04 Nm kg^−1^ (0.01; 0.07)]. Associations between HPA and physical function were observed independent of ST in models 2. In the fully adjusted models (models 3) an additional hour of HPA was associated with longer 6MWD [B = 40.72 m (30.18; 51.25)], TCST_time_ [B = −2.25 s (−3.09; −1.41)], and greater relative elbow flexion strength [B = 0.05 Nm kg^−1^ (0.01; 0.08)] and knee extension strength [B = 0.13 Nm kg^−1^ (0.06; 0.20)].

**Table 3:** **Associations of total physical activity (PA), and higher intensity physical activity (HPA) in hours per day with distance during a six min walk test (6WMD), timed chair rise stand test performance (TCST time), grip strength, elbow flexion strength, and knee extension strength**.

		**Model 1**		**Model 2**		**Model 3**	
		**B**	**95% CI**	**B**	**95% CI**	**B**	**95% CI**
Total PA (h/day)	6 MWT distance (m)	**24.45**	**(19.74; 29.15)**			**16.45**	**(1189; 21.02)**
	TCST time (s)^*^	**−0.88**	**(−1.24;** −**0.52)**			**−0.67**	**(−1.03;** −**0.30)**
	Grip strength (kg kg^−1^)	**0.02**	**(0.01; 0.03)**			0.01	(0.00; 0.01)
	Elbow flexion strength (Nm kg^−1^)	**0.05**	**(0.04; 0.07)**			**0.03**	**(0.01; 0.04)**
	Knee extension strength (Nm kg^−1^)	**0.08**	**(0.05; 0.11)**			**0.04**	**(0.01; 0.07)**
HPA (h/day)	6 MWT distance (m)	**61.25**	**(50.73; 71.77)**	**54.51**	**(43.55; 65.48)**	**40.72**	**(30.18; 51.25)**
	TCST time (s)^*^	**−2.82**	**(−3.62;** −**2.03)**	**−2.58**	**(−3.42;** −**1.75)**	**−2.25**	**(−3.09;** −**1.41)**
	Grip strength (kg kg^−1^)	**0.04**	**(0.03; 0.06)**	**0.03**	**(0.02; 0.05)**	0.01	(0.00; 0.02)
	Elbow flexion strength (Nm kg^−1^)	**0.11**	**(0.08; 0.12)**	**0.09**	**(0.05; 0.13)**	**0.05**	**(0.01; 0.08)**
	Knee extension strength (Nm kg^−1^)	**0.22**	**(0.16; 0.29)**	**0.20**	**(0.13; 0.27)**	**0.13**	**(0.06; 0.20)**

*Results are presented as unstandardized regression coefficients (B) with 95% confidence interval (95% CI)*.

* *Negative coefficient indicates better performance*.

*Associations were adjusted for the following covariates Model 1: waking time, age, sex, type 2 diabetes, and education level. Model 2:Models describing HPA were additionally adjusted for ST (due to collinearity models of total PA were not adjusted for ST). Model 3: model 2 +BMI, alcohol use, smoking status, cardiovascular disease, and health status. Bold fonts indicate statistical significance (p < 0.05)*.

### Additional analyses

To allow a better comparison of the strength of the associations of ST, total PA, and HPA with the physical function outcomes, differences in physical function outcomes were expressed per one standard deviation (*SD*) of ST, total PA, and HPA. Results are presented in Supplemental Table 1 and underline that associations of total PA and HPA with physical function were stronger than associations of ST with physical function.

All analyses were repeated after excluding participants with manifest functional limitations (*n* = 328). The association between ST and 6MWD was attenuated and no longer significant [B = −2.42 (−6.72; 1.86)]. Other results were similar as described above (data not tabulated). Additionally, results were similar after excluding participants with <4 valid days of accelerometer monitoring (*n* = 78).

## Discussion

This study examined the associations of objectivity measured ST, (patterns of) sedentary behavior, PA, and HPA with physical function in a large sample of adults aged 40–75 years. Our results showed that a larger amount of ST was associated with shorter 6MWD, and lower grip strength and elbow flexion strength. Additionally, more sedentary breaks were associated with faster TCST_time_. Longer average sedentary bout duration was associated with slower TCST_time_ and lower knee extension strength. However, the strength of these associations was relatively weak. PA and HPA were associated with greater 6MWD, faster TCSTtime, greater elbow flexion and knee extension strength. The associations of PA and HPA with physical function were stronger than the associations of sedentary behavior variables with physical function.

### Sedentary time

In our study, we observed a weak association between a large amount of ST and lower physical function. Several other epidemiological studies have examined objectively measured ST as a determinant of physical function expressed as gait speed or chair rise test (Santos et al., 2012; Davis et al., 2014; Cooper et al., 2015; Lee et al., 2015; Keevil et al., 2016; Reid et al., 2016; Rosenberg et al., 2016). Findings from these studies were inconsistent as in some studies an association was observed between larger amounts of ST and worse physical function (Santos et al., 2012; Davis et al., 2014; Cooper et al., 2015; Lee et al., 2015; Rosenberg et al., 2016), whereas in other studies no association was observed (Keevil et al., 2016; Reid et al., 2016). To our knowledge, three studies examined associations between objectively measured ST and knee extension strength (Willoughby and Copeland, 2015; Foong et al., 2016; Reid et al., 2016). In agreement with our results, these studies reported no association between ST and knee extension strength. Two other studies reported on associations between objectively measured ST and hand grip strength with different results. Cooper et al. (2015) did observe an association between ST and grip strength, while Keevil et al. (2016) did not. A difference between our study and the others was that we normalized measures of strength for body mass. Normalization for body mass allows better comparisons of strength measures between individuals of different body sizes (Jaric, 2002). We argued that an individual with greater body mass needs more strength to carry his/her own weight, thus a relative measure of strength would better reflect physical function. In addition, compared with absolute measures, normalized measures of hand grip strength and knee extension strength have been associated more strongly with functional limitations (Barbat-Artigas et al., 2013; Dong et al., 2016).

### Patterns of sedentary behavior

In this study we observed some associations between the patterns of sedentary behavior with physical function. However, strength of these associations was rather weak. Few other studies have examined patterns of sedentary behavior and associations with physical function. In a small study (*n* = 44, mean age 70 ± 8 years), Genusso et al. reported that the number of sedentary breaks were positively and the number of prolonged sedentary bouts were negatively associated physical function (Gennuso et al., 2016). In addition, Sardinha et al. reported a positive association between sedentary breaks and physical function in a study with older adults (mean age 73 ± 6 years; Sardinha et al., 2015). In contrast, in the study by Reid et al., sedentary breaks and prolonged sedentary bouts were not associated with physical function (Reid et al., 2016). In our study, which was comparable to the study by Reid et al. in terms of age, we did however observe a small, beneficial association between the number of sedentary breaks and TCST_time_.

Inconsistencies in outcomes between studies may have resulted from a difference in study populations. For example, the study by Reid et al. (2016), who reported no association between sedentary behavior and physical function, had the youngest study population (mean age 58 ± 10 years). The majority of the studies in which a negative association between large amounts of ST and physical function was reported comprised an older population, with mean age >65 years (Santos et al., 2012; Davis et al., 2014; Lee et al., 2015; Rosenberg et al., 2016). A younger population would generally be healthier and have a higher physical functioning. In our study, this was seen by a very high median [25–75%] SF-36 physical function score: 95 [85–100]. Consequently the measures of physical function may have a limited range due to a ceiling effect.

### Physical activity

Positive associations between PA, in particular HPA, and physical function are in line with the literature as summarized in reviews (Paterson and Warburton, 2010; Volkers et al., 2012). Both reviews incorporated longitudinal and/or intervention studies based on self-reported measures of PA. In addition, more recent studies that cross-sectionally examined associations between objectively measured PA and/or MVPA reported a positive association with physical function as well (Santos et al., 2012; Reid et al., 2016). As mentioned, the strength of the associations of sedentary behavior was small compared with the associations of PA and HPA. It is unlikely that the associations of sedentary behavior represent clinically meaningful differences. For example, in a population of COPD patients, ~30 m was found to be the minimal clinically important difference in 6WMD (Polkey et al., 2013). In our study, each additional hour of ST was associated with 2.69 meters shorter 6MWD.

Future studies should examine the associations between objectively measured sedentary behavior and physical function in populations of different ages. Preferably these studies should have a longitudinal design to establish temporality. Importantly, future studies should provide answer to the important question: how much ST is too much? For instance in bed-rest studies, regarded as extreme conditions of ST, substantial muscle mass loss has been observed (Dirks et al., 2016). In our study [and others (Gennuso et al., 2016; Reid et al., 2016)], prolonged bouts of 30 min were used, but perhaps 30 min is not long enough to negatively affect physical function.

### Strengths and limitations

A strength of this study was the use of a posture based accelerometer. The activPAL3 has been found to measure ST and posture transitions (sedentary breaks) more accurately than accelerometers that determine ST based on acceleration data, which have been used in the majority of the studies (Kozey-Keadle et al., 2011; Berendsen et al., 2014). Therefore, estimations of ST were probably more accurate than those in studies using other types of accelerometers. Further, we used multiple objective measures of physical function that reflect upper and lower body function including several measures for muscle strength. However, this study is not without limitations. Importantly, due to the cross-sectional study design, caution is required with regard to causal inferences. It cannot be excluded that due to physical limitations, people engage less time in (H)PA and/or more in sedentary behaviors. However, in additional analyses we have demonstrated that after excluding individuals with mobility limitations the majority of the associations persisted. In addition, step frequency was used to determine HPA. This method may be less precise to determine intensity of PA compared with estimations based on acceleration data. However, we used a step frequency of >110 steps/min which has been reported to correspond to a MET score >3.0 (a commonly used as cut-off value for MVPA). Further, although the actviPAL3 may capture movement and intensity (based on step frequency), it does not provide context of activities. For example, the activPAL3 will classify (strength) training exercises as sedentary when performed in a sitting or lying position. Finally, our study population consisted of a highly functioning population aged 40–75 years. This was partly a result from the exclusion of participants that were unable to perform any of the physical function tests, introducing selection bias. In addition, The Maastricht Study population comprises adults of predominantly Caucasians from European descent. Therefore, generalizability of our results to other populations and ages may be limited. It is not unlikely that associations of sedentary behavior and PA with physical function are different in, for example, frail or institutionalized populations, which have other activity patterns and lower physical function.

## Conclusion

In conclusion, in adults aged 40–75 years, sedentary behavior appeared to be marginally associated with lower physical function, independent of HPA. This suggests that merely reducing sedentary behavior is insufficient to improve/maintain physical function. On the other hand, engaging regularly in PA, and in particular HPA, is important for physical function.

## Author contributions

JvV: Data analysis and writing the first draft of the manuscript. HS, AK, and NS: Study conception, interpretation of the results, and critically reviewed manuscript. JvB: Data acquisition, interpretation of the results, edited, and critically reviewed manuscript. SS, CvK, PD, MS, RH, PR, TvG, and CS: The Maastricht Study design and critically reviewed manuscript. All authors read and approved the final version of the manuscript.

## Funding

This study was supported by the European Regional Development Fund via OP-Zuid, the Province of Limburg, the Dutch Ministry of Economic Affairs (grant 310.041), Stichting De Weijerhorst (Maastricht, the Netherlands), the Pearl String Initiative Diabetes (Amsterdam, the Netherlands), the Cardiovascular Center (CVC, Maastricht, the Netherlands), CARIM School for Cardiovascular Diseases (Maastricht, the Netherlands), CAPHRI Care and Public Health Research Institute (Maastricht, the Netherlands), NUTRIM School for Nutrition and Translational Research in Metabolism (Maastricht, the Netherlands), Stichting Annadal (Maastricht, the Netherlands), Health Foundation Limburg (Maastricht, the Netherlands). Further, the authors declare to have received unrestricted grants from Janssen-Cilag B.V. (Tilburg, the Netherlands), Novo Nordisk Farma B.V. (Alphen aan den Rijn, the Netherlands) and Sanofi-Aventis Netherlands B.V. (Gouda, the Netherlands). None of the funders were involved in the study design or collection, analysis, or interpretation of the data.

### Conflict of interest statement

The authors declare that the research was conducted in the absence of any commercial or financial relationships that could be construed as a potential conflict of interest.

## Abbreviations

PA: physical activity
HPA: higher intensity physical activity
ST: sedentary time
6MWD: distance (meters) covered during the 6 min walk test
TCST_time_: time (in seconds) needed for timed chair rise stand test.

## References

[B1] Barbat-Artigas S. Rolland Y. Cesari M. Abellan Van Kan G. Vellas B. Aubertin-Leheudre M. (2013). Clinical relevance of different muscle strength indexes and functional impairment in women aged 75 years and older. J. Gerontol. A Biol. Sci. Med. Sci. 68, 811–819. 10.1093/gerona/gls254 23262030

[B2] Bauman A. Merom D. Bull F. C. Buchner D. M. Singh M. A. F. (2016). Updating the evidence for physical activity: summative reviews of the epidemiological evidence, prevalence, and interventions to promote “Active Aging.” Gerontologist 56, S268–S280. 10.1093/geront/gnw031 26994266

[B3] Berendsen B. A. Hendriks M. R. Meijer K. Plasqui G. Schaper N. C. Savelberg H. H. (2014). Which activity monitor to use? Validity, reproducibility and user friendliness of three activity monitors. BMC Public Health 14:749. 10.1186/1471-2458-14-749 25059233 PMC4133601

[B4] Brocklebank L. A. Falconer C. L. Page A. S. Perry R. Cooper A. R. (2015). Accelerometer-measured sedentary time and cardiometabolic biomarkers: a systematic review. Prev. Med. 76, 92–102. 10.1016/j.ypmed.2015.04.013 25913420

[B5] Cooper A. J. Simmons R. K. Kuh D. Brage S. Cooper R. Scientific N.. (2015). Physical activity, sedentary time and physical capability in early old age: british birth cohort study. PLoS ONE 10:e0126465. 10.1371/journal.pone.0126465 25961736 PMC4427100

[B6] Cooper R. Kuh D. Cooper C. Gale C. R. Lawlor D. A. Matthews F.. (2011). Objective measures of physical capability and subsequent health: a systematic review. Age Ageing 40, 14–23. 10.1093/ageing/afq117 20843964 PMC3000177

[B7] Cooper R. Kuh D. Hardy R. Mortality Review G. Falcon Teams H. A. S. (2010). Objectively measured physical capability levels and mortality: systematic review and meta-analysis. BMJ 341:c4467. 10.1136/bmj.c4467 20829298 PMC2938886

[B8] Davis M. G. Fox K. R. Stathi A. Trayers T. Thompson J. L. Cooper A. R. (2014). Objectively measured sedentary time and its association with physical function in older adults. J. Aging Phys. Act. 22, 474–481. 10.1123/japa.2013-0042 24085473

[B9] Dirks M. L. Wall B. T. Van De Valk B. Holloway T. M. Holloway G. P. Chabowski A.. (2016). One week of bed rest leads to substantial muscle atrophy and induces whole-body insulin resistance in the absence of skeletal muscle lipid accumulation. Diabetes. 65, 2862–2875. 10.2337/db15-1661 27358494

[B10] Dong R. Wang X. Guo Q. Wang J. Zhang W. Shen S.. (2016). Clinical relevance of different handgrip strength indexes and mobility limitation in the elderly adults. J. Gerontol. A. Biol. Sci. Med. Sci. 71, 96–102. 10.1093/gerona/glv168 26409067

[B11] Evenson K. R. Buchner D. M. Morland K. B. (2012). Objective measurement of physical activity and sedentary behavior among US adults aged 60 years or older. Prev. Chronic Dis. 9:110109. 10.5888/pcd9.110109 22172193 PMC3277387

[B12] Foong Y. C. Chherawala N. Aitken D. Scott D. Winzenberg T. Jones G. (2016). Accelerometer-determined physical activity, muscle mass, and leg strength in community-dwelling older adults. J. Cachexia Sarcopenia Muscle 7, 275–283. 10.1002/jcsm.12065 27239404 PMC4863829

[B13] Gennuso K. P. Thraen-Borowski K. M. Gangnon R. E. Colbert L. H. (2016). Patterns of sedentary behavior and physical function in older adults. Aging Clin. Exp. Res. 28, 943–950. 10.1007/s40520-015-0386-4 26022448

[B14] Jaric S. (2002). Muscle strength testing: use of normalisation for body size. Sports Med. 32, 615–631. 10.2165/00007256-200232100-00002 12141882

[B15] Kahlmeier S. Wijnhoven T. M. A. Alpiger P. Schweizer C. Breda J. Martin B. W. (2015). National physical activity recommendations: systematic overview and analysis of the situation in European countries. BMC Public Health 15:133. 10.1186/s12889-015-1412-3 25879680 PMC4404650

[B16] Keevil V. L. Cooper A. J. Wijndaele K. Luben R. Wareham N. J. Brage S.. (2016). Objective sedentary time, moderate-to-vigorous physical activity, and physical capability in a british cohort. Med. Sci. Sports Exerc. 48, 421–429. 10.1249/MSS.0000000000000785 26501232 PMC4762192

[B17] Kozey-Keadle S. Libertine A. Lyden K. Staudenmayer J. Freedson P. S. (2011). Validation of wearable monitors for assessing sedentary behavior. Med. Sci. Sports Exerc. 43, 1561–1567. 10.1249/MSS.0b013e31820ce174 21233777

[B18] Lee J. Chang R. W. Ehrlich-Jones L. Kwoh C. K. Nevitt M. Semanik P. A.. (2015). Sedentary behavior and physical function: objective evidence from the Osteoarthritis initiative. Arthritis Care Res. (Hoboken) 67, 366–373. 10.1002/acr.22432 25155652 PMC4336845

[B19] Matthews C. E. Chen K. Y. Freedson P. S. Buchowski M. S. Beech B. M. Pate R. R.. (2008). Amount of time spent in sedentary behaviors in the United States, 2003–2004. Am. J. Epidemiol. 167, 875–881. 10.1093/aje/kwm390 18303006 PMC3527832

[B20] Owen N. Healy G. N. Matthews C. E. Dunstan D. W. (2010). Too much sitting: the population health science of sedentary behavior. Exerc. Sport Sci. Rev. 38, 105–113. 10.1097/JES.0b013e3181e373a2 20577058 PMC3404815

[B21] Paterson D. H. Warburton D. E. (2010). Physical activity and functional limitations in older adults: a systematic review related to Canada's Physical Activity Guidelines. Int. J. Behav. Nutr. Phys. Act. 7:38. 10.1186/1479-5868-7-38 20459782 PMC2882898

[B22] Polkey M. I. Spruit M. A. Edwards L. D. Watkins M. L. Pinto-Plata V. Vestbo J.. (2013). Six-minute-walk test in chronic obstructive pulmonary disease: minimal clinically important difference for death or hospitalization. Am. J. Respir. Crit. Care Med. 187, 382–386. 10.1164/rccm.201209-1596OC 23262518

[B23] Reid N. Daly R. M. Winkler E. A. Gardiner P. A. Eakin E. G. Owen N.. (2016). Associations of monitor-assessed activity with performance-based physical function. PLoS ONE 11:e0153398. 10.1371/journal.pone.0153398 27073888 PMC4830578

[B24] Rosenberg D. E. Bellettiere J. Gardiner P. A. Villarreal V. N. Crist K. Kerr J. (2016). Independent associations between sedentary behaviors and mental, cognitive, physical, and functional health among older adults in retirement communities. J. Gerontol. A Biol. Sci. Med. Sci. 71, 78–83. 10.1093/gerona/glv103 26273024 PMC4861254

[B25] Santos D. A. Silva A. M. Baptista F. Santos R. Vale S. Mota J.. (2012). Sedentary behavior and physical activity are independently related to functional fitness in older adults. Exp. Gerontol. 47, 908–912. 10.1016/j.exger.2012.07.011 22884978

[B26] Sardinha L. B. Santos D. A. Silva A. M. Baptista F. Owen N. (2015). Breaking-up sedentary time is associated with physical function in older adults. J. Gerontol. Series A Biol. Sci. Med. Sci. 70, 119–124. 10.1093/gerona/glu193 25324221

[B27] Schram M. T. Sep S. J. van der Kallen C. J. Dagnelie P. C. Koster A. Schaper N.. (2014). The Maastricht study: an extensive phenotyping study on determinants of type 2 diabetes, its complications and its comorbidities. Eur. J. Epidemiol. 29, 439–451. 10.1007/s10654-014-9889-0 24756374

[B28] Sedentary Behaviour Research Network (2012). Letter to the editor: standardized use of the terms “sedentary” and “sedentary behaviours.” Appl. Physiol. Nutr. Metab. 37, 540–542. 10.1139/h2012-02 22540258

[B29] Statistical Office of the European Communities (2015). Functional and Activity Limitations Statistics. Available online at: http://ec.europa.eu/eurostat/statistics-explained/index.php/Functional_and_activity_limitations_statistics Luxembourg, Eurostat (Accessed January 09, 2017).

[B30] Tudor-Locke C. Craig C. L. Brown W. J. Clemes S. A. De Cocker K. Giles-Corti B.. (2011). How many steps/day are enough? For adults. Int. J. Behav. Nutr. Phys. Act. 8:79. 10.1186/1479-5868-8-79 21798015 PMC3197470

[B31] van der Berg J. D. Willems P. J. van der Velde J. H. Savelberg H. H. Schaper N. C. Schram M. T.. (2016). Identifying waking time in 24-h accelerometry data in adults using an automated algorithm. J. Sports Sci. 34, 1867–1873. 10.1080/02640414.2016.1140908 26837855

[B32] Volkers K. M. De Kieviet J. F. Wittingen H. P. Scherder E. J. A. (2012). Lower limb muscle strength (LLMS): why sedentary life should never start? A review. Arch. Gerontol. Geriatr. 54, 399–414. 10.1016/j.archger.2011.04.018 21601928

[B33] Warburton D. E. Charlesworth S. Ivey A. Nettlefold L. Bredin S. S. (2010). A systematic review of the evidence for Canada's physical activity guidelines for adults. Int. J. Behav. Nutr. Phys. Act. 7:1. 10.1186/1479-5868-7-39 20459783 PMC3583166

[B34] Willoughby T. Copeland J. L. (2015). Sedentary time is not independently related to postural stability or leg strength in women 50-67 years old. Appl. Physiol. Nutr. Metab. 40, 1123–1128. 10.1139/apnm-2015-0066 26466084

[B35] Wilmot E. G. Edwardson C. L. Achana F. A. Davies M. J. Gorely T. Gray L. J.. (2012). Sedentary time in adults and the association with diabetes, cardiovascular disease and death: systematic review and meta-analysis. Diabetologia 55, 2895–2905. 10.1007/s00125-012-2677-z 22890825

[B36] World Health Organization (2006). Definition and Diagnosis of Diabetes Mellitus and Intermediate Hyperglycemia: Report of a WHO Consultation. Geneva: World Health Organization.

