# Supplementary material for: Sedentary Behavior Is Only Marginally Associated with Physical Function in Adults Aged 40–75 Years—the Maastricht Study
Source: Front Physiol. 2017 Apr 25;8:242. doi: 10.3389/fphys.2017.00242 (PMC5403943; doi:10.3389/fphys.2017.00242)
Supplement: Supplementary file 1 [file Table1.pdf]

Supplemental Table 1: Differences in distance during a six minute walk test (6WMD), timed chair rise stand test performance (TCST time), grip strength, elbow flexion strength, and knee extension strength expressed per one standard deviation (SD) of sedentary time, total physical activity (PA), and higher intensity physical activity (HPA)

		**Sedentary time**		**Total PA**		**HPA**	
		**B**	**95% CI**	**B**	**95% CI**	**B**	**95% CI**
**Model 1**	6 MWT distance (m)	-12.29	(-15.67; -8.90)	16.54	(13.36; 19.73)	18.47	(15.29; 21.64)
	TCST time (s)*	0.50	(0.24; 0.75)	-0.60	(-0.84; -0.35)	-0.85	(-1.09; -0.61)
	Grip strength (kg kg-1)	-0.01	(-0.02; -0.01)	0.01	(0.01; 0.02)	0.01	(0.00; 0.01)
	Elbow flexion strength (Nm kg-1)	-0.03	(-0.05; -0.02)	0.04	(0.02; 0.05)	0.03	(0.02; 0.05)
	Knee extension strength (Nm kg-1)	-0.04	(-0.06; -0.02)	0.05	(0.03; 0.07)	0.07	(0.05; 0.09)
**Model 2**	6 MWT distance (m)	-7.23	(-10.69; -3.78)			16.44	(13.13; 19.75)
	TCST time (s)*	0.26	(-0.02; 0.52)			-0.78	(-1.03; -0.53)
	Grip strength (kg kg^{-1})	-0.01	(-0.02; -0.01)			0.01	(0.01; 0.02)
	Elbow flexion strength (Nm kg^{-1})	-0.03	(-0.04; -0.01)			0.03	(0.02; 0.04)
	Knee extension strength (Nm kg^{-1})	-0.02	(-0.05; 0.00)			0.06	(0.04; 0.08)
**Model 3**	6 MWT distance (m)	-4.43	(-7.72; -1.14)	11.13	(8.04; 14.22)	12.79	(9.10; 15.45)
	TCST time (s)*	0.19	(-0.08; 0.45)	-0.45	(-0.70; -0.20)	-0.68	(-0.93; -0.42)
	Grip strength (kg kg^{-1})	-0.01	(-0.01; 0.00)	0.00	(0.00; 0.01)	0.00	(0.00; 0.01)
	Elbow flexion strength (Nm kg^{-1})	-0.02	(-0.03; -0.01)	0.02	(0.01; 0.03)	0.01	(0.00; 0.03)
	Knee extension strength (Nm kg^{-1})	-0.01	(-0.03; 0.01)	0.03	(0.01; 0.05)	0.04	(0.02; 0.06)

Results are presented as unstandardized regression coefficients (B) with 95% confidence interval (95% CI). *Negative coefficient indicates better performance. Associations were adjusted for the following covariates Model 1: waking time, age, sex, type 2 diabetes, and education level. Model 2:Models describing ST werte additionally adjusted for HPA. Models describing HPA were additionally adjusted for ST (due to collinearity models of total PA were not adjusted for ST). Model 3: model 2 +BMI, alcohol use, smoking status, cardiovascular disease, and health status. Each unit change (1 SD) corresponds with 1.65 hours for sedentary time, 0.68 hours (41 minutes) for total PA, and 0.30 hours (18 minutes) for HPA.
